# Using metabolite profiling to construct and validate a metabolite risk score for predicting future weight gain

**DOI:** 10.1371/journal.pone.0222445

**Published:** 2019-09-27

**Authors:** Nina Geidenstam, Yu-Han H. Hsu, Christina M. Astley, Josep M. Mercader, Martin Ridderstråle, Maria E. Gonzalez, Clicerio Gonzalez, Joel N. Hirschhorn, Rany M. Salem

**Affiliations:** 1 Clinical Obesity, Institution of Clinical Sciences, Malmö, Lund University, Sweden; 2 Department of Genetics, Harvard Medical School, Boston, MA, United States of America; 3 Division of Endocrinology and Center for Basic and Translational Obesity Research, Boston Children’s Hospital, Boston, MA, United States of America; 4 Programs in Metabolism and Medical & Population Genetics, Broad Institute of Harvard and MIT, Cambridge, MA, United States of America; 5 Diabetes Unit and Center for Genomic Medicine, Massachusetts General Hospital, Boston, MA, United States of America; 6 Instituto Nacional de Salud Publica, Cuernavaca, Morelos, Mexico; 7 Centro de Estudios en Diabetes, Mexico City, Mexico; 8 Department of Family Medicine and Public Health, UC San Diego, San Diego, CA, United States of America; University of Alberta, CANADA

## Abstract

**Background:**

Excess weight gain throughout adulthood can lead to adverse clinical outcomes and are influenced by complex factors that are difficult to measure in free-living individuals. Metabolite profiling offers an opportunity to systematically discover new predictors for weight gain that are relatively easy to measure compared to traditional approaches.

**Methods and results:**

Using baseline metabolite profiling data of middle-aged individuals from the Framingham Heart Study (FHS; n = 1,508), we identified 42 metabolites associated (*p* < 0.05) with longitudinal change in body mass index (BMI). We performed stepwise linear regression to select 8 of these metabolites to build a metabolite risk score (MRS) for predicting future weight gain. We replicated the MRS using data from the Mexico City Diabetes Study (MCDS; n = 768), in which one standard deviation increase in the MRS corresponded to ~0.03 increase in BMI (kg/m^2^) per year (i.e. ~0.09 kg/year for a 1.7 m adult). We observed that none of the available anthropometric, lifestyle, and glycemic variables fully account for the MRS prediction of weight gain. Surprisingly, we found the MRS to be strongly correlated with baseline insulin sensitivity in both cohorts and to be negatively predictive of T2D in MCDS. Genome-wide association study of the MRS identified 2 genome-wide (*p* < 5 × 10^−8^) and 5 suggestively (*p* < 1 × 10^−6^) significant loci, several of which have been previously linked to obesity-related phenotypes.

**Conclusions:**

We have constructed and validated a generalizable MRS for future weight gain that is an independent predictor distinct from several other known risk factors. The MRS captures a composite biological picture of weight gain, perhaps hinting at the anabolic effects of preserved insulin sensitivity. Future investigation is required to assess the relationships between MRS-predicted weight gain and other obesity-related diseases.

## Introduction

Obesity is a global epidemic [1] associated with adverse clinical outcomes including type 2 diabetes (T2D) [2], cardiovascular disease (CVD) [3], non-alcoholic fatty liver disease [4], and death [5]. Population studies demonstrate that adults, on average, continue to gain weight at a rate of 0.4–1 kg per year through the middle decades of life [6–8]. Likewise, the percentage of adults who are overweight or obese (body mass index, BMI ≥ 25 kg/m^2^) has increased from around 29% to 37% globally over the past three decades [1].

Excess weight gain can be intuitively explained by an excess of energy intake relative to energy expenditure and energy partitioning within an individual; however, the components leading to energy imbalance are very complex, highly interactive, and hard to measure in free-living populations over extended time periods [9]. Therefore, even though studies have identified various risk factors for weight gain, such as unhealthy diet, reduced physical activity, change in smoking status, and glucose-stimulated insulin secretion, the biological mechanisms linking these risk factors to weight gain have yet to be fully elucidated [8,10–12]. In terms of genetic predisposition, while recent genome-wide association studies (GWAS) have identified hundreds of BMI-associated loci [13,14], little is known about the genetics of longitudinal change in BMI, which do not always agree with that of cross-sectional BMI [15]. Furthermore, while recent studies have developed multivariate models that incorporate different types of risk factors to predict weight gain [16,17], these models often include variables that may not be readily available in many research or clinical settings (e.g. recalled lifestyle and dietary variables derived from detailed questionnaires and specialized calculations). Overall, the etiology of obesity remains incompletely understood and there is a lack of easily generalizable prediction models for weight gain, thus non-surgical interventions to modify weight have largely been met with modest effect [18,19].

Metabolite profiling systematically measures metabolite levels in biological samples and offers a new, powerful approach for studying both intrinsic (i.e. genetic) and extrinsic (i.e. environmental) influences on obesity phenotypes in a data-driven, hypothesis-free manner that does not depend on prior knowledge. Novel biomarkers for a trait could be identified by testing for correlation between metabolites and the trait measured at a single time point; furthermore, if longitudinal data are available, predictive biomarkers could also be identified by associating metabolites with a future outcome. Numerous studies have successfully applied this approach to identify correlated or predictive biomarkers for obesity and cardiometabolic traits [20–27]. For instance, branched-chain amino acids (BCAAs), aromatic amino acids, and lipids with low carbon number and double bond content have been shown to correlate with cross-sectional BMI [21,22] and even predict the risk of developing insulin resistance and/or T2D [24–26].

In contrast, there are limited studies of metabolite profiling with longitudinal weight trajectories. A few studies have identified lipids, BCAAs, and metabolites involved in energy metabolism and/or oxidative stress to be correlated with past weight change [28,29]. While these correlated biomarkers provide insights into the metabolic consequences of weight change, they are not as useful as predictive biomarkers in terms of informing clinical obesity prevention strategies. On the other hand, studies that looked at association between metabolites and future weight change have identified acetylcholine, leucine, hippuric acid, acetylglycine, urate, and xanthine to be predictive biomarkers for significant weight gain [29,30]. These metabolites may be used as clinical risk factors and may even point to causal biological pathways for weight gain. However, none of these studies address how the individual metabolites may be combined into a replicable and generalizable risk score for predicting weight gain, nor do the studies compare the metabolites against conventional obesity risk factors to evaluate their independence and predictive power. These additional analyses are important for assessing the potential usefulness of metabolite predictors for weight gain.

The objectives of this study were to construct and validate a metabolite risk score (MRS) consisting of multiple metabolites for predicting future weight gain and to investigate the underlying biology captured by the MRS. Towards this end, we used longitudinal BMI measurements and baseline metabolite profiling data of 1,508 middle-aged individuals from the Framingham Heart Study (FHS) to construct an MRS consisting of 8 metabolites. The association of the MRS with future weight gain was replicated using data from 768 individuals in the Mexico City Diabetes Study (MCDS), demonstrating its validity and generalizability across study populations. To understand the biological information contained within the MRS, we investigated its association with other obesity-associated factors and performed a GWAS for the MRS. Our findings indicate that the components of the MRS reflect diverse biological factors related to weight gain, possibly including anabolic effects of preserved insulin sensitivity and protection from T2D, which are unlikely to be captured by single metabolites or other obesity-associated factors.

## Subjects and methods

### Study samples and datasets

Discovery cohort: Data for the Framingham Heart Study (FHS), a prospective cohort study of CVD risk factors, were obtained through the NCBI database of Genotypes and Phenotypes (dbGaP) with accession number phs000007.v19.p7 [24,31]. Metabolite profiling was performed on 2,016 fasting plasma samples from the FHS Offspring Cohort collected at Exam 5 (1991–1995) using liquid chromatography-mass spectrometry (LC-MS) [32]. We filtered samples with quality controlled metabolite data (n = 2,015; see *Data processing*) to exclude samples with missing BMI values at Exam 5 or Exam 7 (1998–2002) (n = 271) and samples with clinical diagnosis of T2D (n = 141), CVD (n = 154) and/or renal disease (n = 53) at Exam 5, resulting in a total of 1,508 samples. T2D was defined as having fasting plasma glucose ≥ 126 mg/dl or using insulin or other anti-diabetic medications; CVD and renal disease status were ascertained using ‘Survival File and Follow-up for Cardiovascular Events’ (pht003316) and Exam 5 clinical exam (pht000034). For genome-wide association analyses, we further restricted to a subset of 1,349 samples with available genetic data (see **S1 Text** for details).

Replication cohort: The Mexico City Diabetes Study (MCDS), a prospective cohort study for T2D, has been described previously [33,34]. Briefly, LC-MS profiling was performed on 865 fasting plasma samples collected at the 1998 visit. Starting with 841 samples with quality controlled metabolite data (see *Data processing*), we excluded one sample with incorrect height measurement, samples with missing BMI values at the 1998 or 2008 visit (n = 3), and samples with CVD (n = 68) or renal disease (n = 1) at the 1998 visit, resulting in 768 samples for use in analysis. No T2D cases were present at the 1998 visit. T2D was defined as having fasting glucose ≥ 126 mg/dl, 2-hour glucose ≥ 200 mg/dl in oral glucose tolerance test, or using anti-diabetic medication; CVD was defined as receiving a possible or probable myocardial infarction diagnosis in electrocardiograph results; renal disease was defined as having history of receiving kidney dialysis, transplant, or medication. For genome-wide association analyses, we used a subset of 588 samples with available genetic data (see **S2 Text** for details).

Data collection for both cohorts were conducted at external facilities by researchers not involved in the design and analysis of this study. All participants provided informed consent. This study was approved by the Boston Children’s Hospital and UC San Diego Institutional Review Boards.

### Data processing

Change in BMI: The rate of change in BMI was calculated as difference in BMI divided by difference in age between the baseline (*b*) and follow-up (*f*) time points: (*BMI*_*f*_−*BMI*_*b*_)/ (*Age*_*f*_*−Age*_*b*_), with age expressed in years. For FHS, we used Exams 5 and 7 as the baseline and follow-up time points, respectively; for MCDS, we used the 1998 and 2008 visits as the two time points. Height and weight values measured during the exam visits were used to derive BMI at each time point for both cohorts. We adjusted the rate of change in BMI for baseline age and sex and refer to the adjusted values as ΔBMI. As a sensitivity analysis, we performed rank-based inverse normal transformation on ΔBMI and confirmed that the transformed vs. untransformed ΔBMI values produced comparable results. To retain interpretability, we used the untransformed ΔBMI in units of kg/m^2^/yr in downstream analyses.

Metabolite data: Baseline metabolite profiling data for each cohort were quality controlled and standardized using the following steps: (1) remove samples and metabolites with > 25% missing data, (2) log-transform the remaining metabolites, (3) adjust for covariates (age, sex, and fasting time for FHS; age and sex for MCDS), (4) impute missing values using a multiple imputation approach implemented in the MICE R package (v2.25; see **S3 Text** for details) [35], and (5) perform rank-based inverse normal transformation to calculate metabolite abundance *z*-scores. After these steps, we limited all subsequent analyses to 183 metabolites shared between the FHS and MCDS datasets.

Genetic risk score for BMI: We used 97 previously identified BMI-associated variants [13] and effect size estimates for their BMI-increasing alleles to calculate an effect size-weighted genetic risk score (GRS_BMI_) for both FHS and MCDS samples. Large-scale Mexican-based BMI GWAS was not available, so European-based effect estimates were used for both cohorts despite ancestry differences.

Smoking status: Self-reported smoking information was available for FHS at both baseline and follow-up time points. We constructed a binary “Quit Smoking” variable, where subjects that were smokers at baseline but not at follow-up were categorized as “true” (n = 97) and subjects whose smoking status did not change were categorized as “false” (reference category).

Physical activity: Physical activity (PA) was calculated as the sum of time spent on moderate and heavy physical activities per day (hours/day), using self-reported questionnaires. PA data were not available at baseline, so we used the PA data collected prior to baseline time points, i.e. Exam 4 (mean 3.65 years before baseline) for FHS and the 1991 visit (mean 6.30 years before baseline) for MCDS. For FHS, which had follow-up PA data, we were able to calculate change in PA (ΔPA) as PA at Exam 7 minus PA at Exam 4.

Dietary intake: Daily macronutrient values (grams/day) derived from self-reported food frequency questionnaires were used to calculate total caloric intake (calories/day) and the percentage of energy intake from carbohydrate (Carb Intake), fat (Fat Intake), and protein (Protein Intake). For FHS, dietary data collected at baseline were used; for MCDS, data collected at the 1991 visit were used due to lack of data available at baseline. For FHS, dietary fat intakes (monounsaturated, polyunsaturated, and saturated fats; grams/day) were also available.

Glycemic measures: Plasma fasting glucose (mg/dl) and insulin (mIU/l) concentrations, as well as 2-hour concentrations after oral glucose tolerance test, were available at baseline for both cohorts. The homeostasis model assessment of insulin resistance (HOMA-IR), Matsuda insulin sensitivity index (Matsuda ISI), and quantitative insulin sensitivity check index (QUICKI) were also available and calculated as described previously [36–38].

### Constructing the MRS for weight gain

A schematic overview of the protocol for constructing, validating, and evaluating the MRS is provided in **S1 Fig**.

Identifying ΔBMI-associated metabolites (**S1A(i) Fig**): First, to identify metabolites associated with ΔBMI, we performed univariate linear regression of ΔBMI on each metabolite in FHS. As both ΔBMI and metabolites were pre-adjusted for covariates (see *Data processing* above), no additional variables were included in the regression models. Since FHS contains families of related individuals (**S2 Fig**), we also performed sensitivity analysis using a linear mixed model approach implemented in the lmerTest R package (v2.0–29) to account for sample relatedness as estimated by pedigree. We observed that the association statistics obtained using simple linear regression vs. linear mixed model were highly consistent (**S1 Table** and **S3 Fig**), and therefore used linear regression in all subsequent analyses. For metabolites that showed nominally significant (*p* < 0.05) association, we tested for their replication in MCDS (although this replication was not required for including metabolites in the MRS as described below). Clustered metabolite correlation heat maps were created using FHS data and the heatmap.2() function in the gplots R package (v3.0.1).

Building the MRS model (**S1 A(ii) Fig**: To build an optimal multivariate model for predicting weight gain using FHS data, metabolites nominally associated (*p* < 0.05) with ΔBMI in FHS were used as input variables into a stepwise regression procedure implemented in the step() function in R (v3.2.1). In order to determine which direction of variable selection (forward, backward, or bidirectional) and model evaluation metric (Akaike information criterion, AIC or Bayesian information criterion, BIC) to use for running the stepwise procedure, we performed 100 repeated 10-fold cross validations (**S4 Fig**). Based on the cross-validation results, the AIC metric combined with a bidirectional selection approach was used to train the final stepwise model using all FHS data. We estimated partial R^2^ of each metabolite (i.e. proportion of variance uniquely explained by the metabolite) in the final model by comparing the full model against a reduced model without the metabolite: *partial R*^*2*^ *= (SSE*_*reduced*_*−SSE*_*full*_*)/SSE*_*reduced*_, *where SSE = error sum of squares*.

Calculating the MRS *z*-scores (**S1A(iii) and S1B(i) Fig**): We calculated a single MRS value for each subject in either FHS or MCDS as: Σ*β*_*i*_ *z*_*i*_, where *β*_*i*_ is the effect size estimate for each metabolite in the FHS stepwise model and *z*_*i*_ is the corresponding metabolite abundance *z-*score for the sample. Rank-based inverse normal transformation was performed on the MRS values for each cohort and the resulting MRS *z*-scores were used for all subsequent analyses.

### Association analyses for ΔBMI, MRS, and other obesity-related phenotypes

Linear regression was performed to test for association between ΔBMI, MRS (or individual metabolites in the MRS), and other obesity-related variables, including baseline BMI and waist-to-hip ratio (WHR), GRS_BMI_, smoking status, physical activity, dietary intakes, and glycemic measures (**S1A(iv), S1B(ii) and S1C(i) Fig**). Logistic regression was performed to associate future T2D status with MRS, baseline anthropometric (BMI and WHR), or glycemic measures (**S1C(ii) Fig**). When testing for independence of MRS and another variable in terms of association with ΔBMI, we included the variable as a covariate in regression analysis to see whether it would affect the statistical significance of the association observed between ΔBMI and MRS. When applicable, we estimated partial R^2^ of each variable (i.e. proportion of variance uniquely explained by the variable) in ΔBMI multivariate models, using the same formula described for the MRS model above.

### GWAS for MRS and MRS metabolites

Genome-wide association analyses were performed in both FHS and MCDS to identify genetic loci associated with MRS and with the individual metabolites in the MRS (**S1C(iii) Fig**). To account for sample relatedness, we used the linear mixed model and kinship matrix calculation methods in EPACTS (v3.2.6) [39] to perform the GWAS in each cohort. For MCDS, we also included genotyping platform as a covariate (since genotype imputation was performed separately for each platform). We separately analyzed genetic variants with minor allele count > = 3 in FHS or MCDS, and meta-analyzed the two cohorts using the inverse variance weighted method in METAL (2011-03-25 version) [40]. To identify independent loci, we “clumped” the meta-analysis results, grouping together sets of nearby, correlated variants that are within 500kb and have pairwise *r*^2^ > 0.1. We used PLINK (v1.9) [41] and 1000 Genomes phase 1 reference panel [42] to generate the clumps of correlated variants and selected the “lead” (best-associated) variant from each clump. We used the Variant Effect Predictor [43] to identify nearest genes (± 5kb) for the lead variants. For MRS-associated lead variants reaching suggestive significance (*p* < 1 × 10^−6^), we also searched the NHGRI-EBI GWAS catalog (v1.0.2, 2019-05-03) [44] for traits previously associated (*p* < 5 × 10^−8^) with the variants (± 50kb).

## Results

### Metabolites associated with longitudinal change in BMI

**Fig 1:**
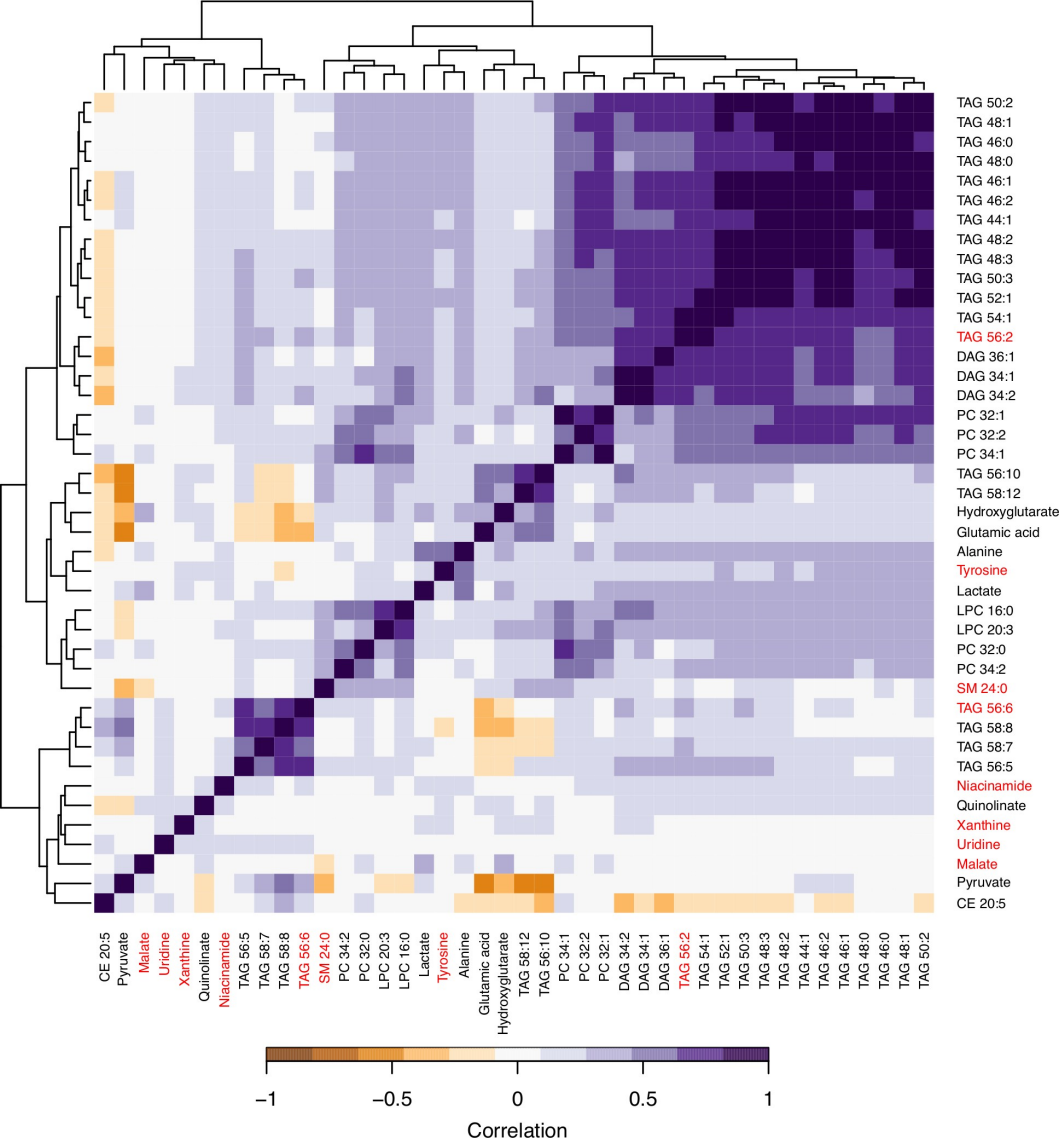
Clustered correlation heat map of the 42 ΔBMI-associated (*p* < 0.05) metabolites in FHS. The 8 metabolites included in the MRS are labeled in red. TAG, triacylglyceride; DAG, diacylglyceride; PC, phosphatidylcholine; LPC, lysophosphatidylcholine; SM, sphingomyelin; CE, cholesteryl ester.

We used longitudinal BMI measurements to calculate age- and sex-adjusted change in BMI over time (ΔBMI) for 1,508 samples from FHS (demographic and clinical characteristics shown in **Table 1**). The mean ΔBMI is 0.127 kg/m^2^/year (standard deviation, SD = 0.320) with mean follow-up interval of 6.9 years. Using baseline metabolite profiling data, we identified 42 metabolites that have nominally significant association (*p* < 0.05) with ΔBMI (**S1 Table**). Increased levels of 35 metabolites are associated with less weight gain (i.e. lower ΔBMI). These include tyrosine, malate, alanine, niacinamide, hydroxyglutarate, glutamic acid, xanthine, quinolinate, lactate, many lipids with relatively lower double bond content (i.e. between 0–3 double bonds; except for 2 triacylglycerides, TAG 56:10 and 58:12). On the other hand, 7 metabolites show the opposite trend, where increased levels are associated with more weight gain (i.e. higher ΔBMI). These include uridine, pyruvate, and 5 lipids with higher double bond content (TAG 56:6, 56:6, 58:7, 58:8, and a cholesteryl ester, CE 20:5). Overall, the ΔBMI-associated metabolites are spread out in different correlation clusters among all tested metabolites, with lipids of similar saturation level (i.e. double bond content) showing especially strong correlation with each other (**Fig 1 and S5 Fig**).

**Table 1:** Descriptive characteristics of the study cohorts.

Characteristics	Framingham Heart Study (FHS)		Mexico City Diabetes Study (MCDS)	
Number of samples	1508		768	
Number of female samples	804 (53.3)		470 (61.2)	
ΔBMI (kg/m^2^/yr)	0.127 ± 0.320		0.000 ± 0.220	
**Characteristics by Time Point**	**Baseline** **(Exam 5)**	**Follow-up** **(Exam 7)**	**Baseline** **(1998 visit)**	**Follow-up** **(2008 visit)**
Age (yr)	54.1 ± 9.52	61.0 ± 9.43	52.2 ± 7.59	62.4 ± 7.56
BMI (kg/m^2^)	27.4 ± 4.87	28.3 ± 5.33	28.5 ± 4.43	29.5 ± 4.80
Fasting Glucose (mg/dl)*	94.4 ± 9.46	97.8 ± 10.1	90.7 ± 10.1	99.7 ± 32.2
T2D	0 (0)	105 (6.96)	0 (0)	144 (18.8)

Showing mean ± standard deviation for quantitative traits or sample count (%) for binary traits.

* Fasting glucose: excluded T2D cases in both cohorts and missing measurements in MCDS (n = 16).

### MRS model for weight gain

In order to combine the predictive power of individual metabolites, we performed stepwise regression using the 42 ΔBMI-associated metabolites in FHS to build an MRS model for weight gain. The resulting stepwise model includes eight metabolites that each contributes to the prediction of weight gain: TAG 56:6, malate, niacinamide, sphingomyelin (SM) 24:0, uridine, TAG 56:2, tyrosine, and xanthine (**Table 2**). These metabolites show relatively weak correlation with each other (*r* = -0.097 to 0.319, **S2 Table** and **S6 Fig**) and represent distinct clusters formed by the 42 ΔBMI-associated metabolites (**Fig 1**). Using these 8 metabolites in the stepwise model, we calculated an effect size-weighted MRS score for all FHS samples and observed that this MRS is strongly associated with ΔBMI (β = 0.0612, *p* = 1.82 × 10^−14^) and explains 3.83% of the observed ΔBMI variance (**S3 Table** and **S7 Fig**). However, this estimate is likely inflated due to overfitting and the winner’s curse [45], as indicated by our cross validation results (i.e. MRS models generated using 10-fold cross validations all had R^2^ < 0.383; **S4 Fig**).

**Table 2:** Metabolite risk score model for predicting weight gain in FHS.

Metabolite	β	SE	CI 2.5	CI 97.5	P-value	Partial R^2^
TAG 56:6	0.0310	0.0088	0.0138	0.0482	4.17E-04	0.0083
Malate	-0.0241	0.0082	-0.0401	-0.0080	3.27E-03	0.0058
Niacinamide	-0.0226	0.0084	-0.0389	-0.0062	6.98E-03	0.0048
SM 24:0	-0.0209	0.0081	-0.0367	-0.0050	9.88E-03	0.0044
Uridine	0.0211	0.0084	0.0046	0.0375	1.20E-02	0.0042
TAG 56:2	-0.0203	0.0089	-0.0378	-0.0027	2.38E-02	0.0034
Tyrosine	-0.0177	0.0084	-0.0342	-0.0013	3.49E-02	0.0030
Xanthine	-0.0145	0.0082	-0.0306	0.0017	7.88E-02	0.0021

β, effect size estimate; SE, standard error of β; CI 2.5—CI 97.5, 95% confidence interval for β; Partial R^2^, proportion of variance uniquely explained by the metabolite (see Methods); TAG, triacylglyceride; SM, sphingomyelin.

### Replication in an independent cohort

To replicate the associations of individual metabolites and the MRS in an independent cohort, and to obtain unbiased estimates of effect size, we first analyzed the 42 ΔBMI-associated metabolites in 768 samples from MCDS. The mean ΔBMI in MCDS is 0 kg/m^2^/year (SD = 0.320; mean follow-up interval = 10.7 years; **Table 1**), which does not differ significantly from that of FHS (*p* = 0.0913 in Welch’s two-sample *t*-test). In MCDS, 36 of the 42 (85.7%) metabolites show directionally consistent ΔBMI associations compared to FHS, with 24 (57.1%) also reaching nominal significance, providing strong evidence of replication in an independent cohort drawn from a different population (**S1 Table**). Next, we calculated the effect size-weighted MRS score for all MCDS samples using the FHS stepwise model and validated that the MRS significantly predicts weight gain in MCDS (β = 0.0298, *p* = 1.63 × 10^−4^; **S3 Table** and **S7 Fig**), where 1.84% of ΔBMI variation is explained by the score. The observed effect size estimate indicates that one SD increase in MRS predicts 0.0298 kg/m^2^/year increase in ΔBMI (i.e. ~0.9 kg over 10 years for a 1.7 m adult).

### Anthropometric and lifestyle risk factors do not mediate prediction of ΔBMI by the MRS

**Fig 2:**
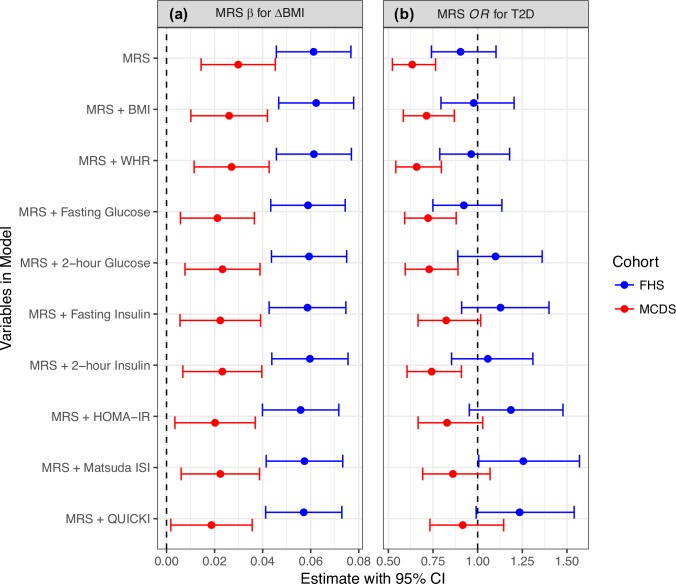
Association between ΔBMI or future T2D status and MRS with adjustment for baseline anthropometric or glycemic measures. **(a)** Effect size estimates (β) of MRS for ΔBMI. **(b)** Odds ratios (OR) of MRS for T2D status at follow-up. Variables in Model, which variables were included in the linear (for ΔBMI) or logistic (for T2D) regression model when estimating the effect of MRS. Error bars indicate 95% confidence interval (CI). Vertical dashed black lines indicate null effect (β = 0 or OR = 1).

We investigated the relationships between ΔBMI, MRS, and various obesity-related risk factors, both to gain insights regarding the underlying biology captured by the MRS and to evaluate whether the MRS is independent of these factors. First, we assessed two baseline anthropometric measures: BMI and WHR. Both measures are inversely correlated with ΔBMI in MCDS (but show no association in FHS) and with MRS in both cohorts (**S4 Table**). However, conditional analyses using BMI or WHR as covariate showed that the association between ΔBMI and MRS remains strongly significant after adjusting for either variable (**Fig 2A** and **S3 Table**). We also asked whether a genetic risk score for cross-sectional BMI (GRS_BMI_, see Methods) could predict ΔBMI. As expected, GRS_BMI_ is significantly associated with baseline age and sex-adjusted BMI in both cohorts (*p* = 2.37 × 10^−6^ in FHS; *p* = 5.51 × 10^−3^ in MCDS); the degree of association is similar between FHS and MCDS (i.e. overlapping 95% confidence intervals for β; **S4C Table**) despite differences in ancestry. However, GRS_BMI_ is not associated with ΔBMI or with MRS (**S4 Table**). In summary, none of the anthropometric-related risk factors we tested account for the predictive power of MRS for weight gain, even though the MRS can be associated with baseline BMI and WHR.

We also looked at several lifestyle factors that can influence weight gain: smoking status, physical activity, and dietary intake measures. Quitting smoking is strongly associated with ΔBMI but not with MRS (**S4 Table**), and adjusting for this variable did not affect the MRS prediction of ΔBMI (**S3 Table**). To further assess confounding due to smoking, we performed a sensitivity analysis by regressing ΔBMI against MRS using only baseline non-smokers in FHS. The association between MRS and ΔBMI in non-smoking samples is highly significant (n = 1,243, β = 0.045, *p* = 1.96 x 10^−7^, R^2^ = 0.021), albeit with a slightly diminished effect size estimate (empirical *p* < 0.0061, calculated using 10,000 random samplings of 1,243 FHS samples) compared to association in all samples (n = 1,508, β = 0.055, *p* = 5.80 x 10^−12^, R^2^ = 0.031). Overall, these results indicate that while smoking is associated with weight gain in our data, it does not explain the observed association between ΔBMI and MRS.

In contrast to smoking status, neither physical activity nor dietary intake variables are significantly correlated with ΔBMI and MRS in our data (**S4 Table**). Furthermore, based on results of conditional analyses, none of these variables account for the association between ΔBMI and MRS (**S3 Table**). More detailed analysis of the FHS dietary data, however, showed that several MRS metabolites are nominally associated with different types of fat intake (TAG 56:6 with saturated and monounsaturated fats, malate with polyunsaturated fat, uridine with saturated and polyunsaturated fats, and xanthine with saturated fat; **S5 Table**), suggesting that these metabolites may be influenced by dietary patterns. In general, our results demonstrate that the MRS is a stronger predictor for weight gain compared to lifestyle factors that measure energy intake (i.e. diet) or expenditure (i.e. physical activity) in our datasets, and the MRS has the additional advantage of not relying on participant recall.

### Insulin resistance measures and future T2D outcome are negatively associated with MRS

Next, to better understand the physiological correlates of the information contained within the MRS, we tested whether baseline clinical measures of dysglycemia and insulin resistance are associated with ΔBMI and MRS. In both FHS and MCDS, ΔBMI and MRS are generally negatively correlated with variables that assessed insulin resistance (i.e. fasting/2-hour glucose and insulin, and HOMA-IR) and positively correlated with those that marked insulin sensitivity (i.e. Matsuda ISI and QUICKI) (**S4 Table**). Analysis of individual metabolites within the MRS indicated that TAG 56:2 and tyrosine are the strongest drivers of the associations between MRS and glycemic response in both cohorts, with the remaining metabolites showing less consistent associations (**S6 Table**). The percentage of ΔBMI variance explained by the MRS (1.84% in MCDS) is on the same order of magnitude as the percentage explained by the various glycemic traits (1.38 to 5.21%; **S4 Table**) and BMI GRS. In conditional analyses, the relationship between ΔBMI and MRS remained nominally significant after adjusting for each of the glycemic variables (**Fig 2A** and **S3 Table**), implying that the MRS is not solely a proxy for insulin sensitivity.

In both FHS and MCDS, the baseline anthropometric and glycemic measures are, as expected, significantly associated with future incident T2D (*p* = 1.93 × 10^−29^ to 1.10 × 10^−3^; **S7 Table**). Therefore, we tested whether the MRS can be used to predict future T2D using logistic regression and assessed whether its predictive power is independent from these baseline variables. Unexpectedly, the MRS is negatively associated (*p* = 2.57 × 10^−6^) with T2D in MCDS, where one SD increase in MRS translates into 36.6% reduction in risk of developing T2D over a ~10-year period; in FHS, the MRS also has a negative but nonsignificant association with T2D (**Fig 2B** and **S8 Table**). However, when we included specific glycemic variables (i.e. fasting insulin, HOMA-IR, Matsuda ISI, and QUICKI) as covariates during conditional analyses, the association between T2D status and MRS in MCDS became nonsignificant (*p* > 0.05; **Fig 2B** and **S8 Table**). Thus, measured baseline glycemic variables could account for the observed association between MRS and lower incident T2D, but not continued rise in BMI as shown in **Fig 2A**.

### Genetic loci associated with MRS

Finally, we utilized genetic data to begin to dissect the underlying biology reflected by the MRS. We performed separate GWAS for the MRS in FHS and MCDS and meta-analyzed the association results. We identified 2 genome-wide significant loci (*p* < 5 × 10^−8^; **Table 3**). The most significant locus (rs174565; *p* = 5.97 × 10^−10^) lies in the *FADS1/FADS2* gene cluster, both of which encode fatty acid desaturases that introduce double bonds into fatty acids [46]. This locus has been associated with a wide range of traits (**S9 Table**), including lipid-related metabolites [47,48], fasting glucose [49], liver enzyme levels [50], and heart function traits [51–53]. The second locus (rs192386132; *p* = 2.80 × 10^−8^) is near *TACC2*, which encodes a transforming acidic coiled-coil containing protein that has been linked to cancer [54]; no previous GWAS associations have been reported near (± 50kb) this locus. We also identified five additional loci at suggestive significance (*p* < 1 × 10^−6^; **Table 3**), 2 of which overlap with BMI loci reported in the GWAS catalog (rs12325540 and rs146167165; **S9 Table**).

**Table 3:** Genetic loci reaching suggestive significance (*p* < 1 × 10^−6^) in MRS GWAS meta-analysis.

Variant ID	Chr	Position	EA	OA	EAF	β	SE	P-value*	R^2^	Nearest Gene (± 5kb)	In GWAS Catalog
rs174565	11	61591636	C	G	0.701	0.267	0.043	**5.97E-10**	0.030	*FADS1*, *FADS2*	Yes
rs192386132	10	123937855	A	G	0.003	1.981	0.357	**2.80E-08**	0.025	*TACC2*	No
rs12325540	16	73380363	C	T	0.788	0.236	0.044	8.63E-08	0.019	*-*	Yes
rs72848293	17	72314849	A	G	0.052	0.444	0.087	3.17E-07	0.019	*DNAI2*	No
rs113940640	9	37228390	A	T	0.023	0.657	0.130	4.33E-07	0.019	*ZCCHC7*	Yes
rs146167165	6	163054293	T	C	0.013	0.986	0.197	5.26E-07	0.024	*PARK2*	Yes
rs188635171	12	7378287	A	G	0.009	1.087	0.219	7.07E-07	0.020	*-*	Yes

* Genome-wide significant *p*-values are in bold. Chr, chromosome; Position, hg19 genomic position; EA, effect allele (i.e. MRS-increasing allele); OA, other allele; EAF, effect allele frequency; β, effect size estimate; SE, standard error of β; R^2^, proportion of variance explained, estimated as: β^2^ × 2f(1-f), where f = EAF; In GWAS Catalog, whether the locus (± 50kb) has been linked to traits in the GWAS catalog (see **S9 Table** for list of associated traits).

Since the MRS is a composite phenotype generated using 8 individual metabolites, we also performed GWAS separately for each of the metabolites and identified a total of 37 loci reaching at least suggestive significance (**S10 Table**). Only 3 of these loci are genome-wide significant (2 for TAG 56:6 and 1 for TAG 56:2), one of which overlaps with the *FAD1/FADS2* locus for MRS. Interestingly, the *TACC2* locus, which is genome-wide significant for MRS, does not show up as significant for any of the MRS metabolites. Together, these findings suggest that while the MRS can reflect biology driven by individual metabolites in the MRS (e.g. TAG 56:6), it might also be capturing more complex biological patterns that are not specific to a single metabolite.

## Discussion

In this study, we defined a metabolite risk score (MRS) incorporating eight individual metabolites, which predicts change in BMI over a span of 7–10 years. This MRS was constructed and validated using two independent cohorts with demographic differences (e.g. geography, ancestry, lifestyle, and dietary patterns), indicating the generalizability of the score. Based on our results, we estimated that adults of average height (~1.6 m for women to ~1.8 m for men) with high MRS (+1 SD above mean) would gain about 0.15 to 0.19 kg more than those with low MRS (-1 SD below mean) per year. Given that the average person gains 0.4–1.0 kg/year in adulthood, identifying individuals with high-risk MRS could be useful on both the individual and population levels, especially if additional metabolites are identified to improve predictive power. Importantly, none of the routinely measured anthropometric, lifestyle, and glycemic risk factors we tested fully account for the association between ΔBMI and MRS. The proportion of ΔBMI variance explained by the MRS is comparable to that for the best predictive risk factors (i.e. glycemic measures). Therefore, the MRS can be used as an independent predictor for studying weight gain.

The MRS consists of eight metabolites, including three lipids (TAG 56:6, SM24:0, and TAG 56:2), malate, niacinamide, uridine, tyrosine, and xanthine, and may point to independent biological processes related to weight gain. For the lipids, we observed that TAG 56:6 is positively correlated with ΔBMI, while SM 24:0 and TAG 56:2, which have lower double bond content, show the opposite trend. This pattern is in accordance with previous studies that found lipids with varying saturation levels to have opposite association with obesity-related phenotypes. For instance, lipids of lower vs. higher double bond content were previously associated with increased vs. decreased risk of diabetes in FHS, respectively [25]. Here, we observed that TAG 56:2 and SM 24:0 are linked to decreased weight gain in both FHS and MCDS and increased T2D risk in MCDS (i.e. they have negative effect sizes in the MRS that is protective against diabetes), while TAG 56:6 has the opposite direction of effect. Among the non-lipid metabolites, xanthine has been previously identified as a predictor for significant future weight gain [30]; and uridine is closely related to both xanthine and urate [55], which was another predictor identified in the same study. Niacinamide is involved in nicotinamide adenine dinucleotide biosynthesis and has been connected to obesity and weight regulation through its role in energy metabolism [56,57]. Tyrosine has been associated with past weight gain, insulin resistance, dyslipidemia, and T2D [24,26,29,58]. Finally, though malate has not been linked directly to weight gain, it is involved in several metabolic pathways that are essential for energy metabolism, including the TCA cycle, pyruvate/malate-cycle, and malate/aspartate shuttle [59,60]. Further investigation is necessary to test whether these MRS metabolites exert causal influence on weight gain.

When we investigated whether the MRS might be tagging specific aspects of obesity-related physiology, we found it to be a strong marker of preserved insulin sensitivity. This finding is consistent with the notion that insulin effect can drive obesity and agrees with previous studies that found insulin to be associated with increased weight gain [61,62]. The unanticipated negative correlations between the MRS and baseline insulin resistance or future T2D in our data have important implications for interpreting our results: historically, excess weight (or BMI) has been used as a proxy for metabolic dysfunction associated with disease outcomes, under the assumption that higher weight gain is associated with higher risk of developing diseases; however, part of our MRS actually captures a “healthy” component of weight gain with respect to diabetes risk and identified weight-gainers who had more robust insulin secretion. Thus, for clinical disease prevention efforts related to T2D, it might be more fruitful to target individuals with low MRS who are less likely to gain weight but are more metabolically unhealthy with respect to T2D. Future investigation is needed to test if this type of inverse association also exists between the MRS and other obesity-related diseases such as CVD, liver disease, cancer and mortality. In any case, we note that the MRS-T2D association we observed loses statistical significance after adjustment for some of the glycemic measures, indicating that the MRS and these measures are capturing the same insulin effect signature responsible for T2D prediction. In contrast, the MRS-ΔBMI association appears to be partially independent from insulin effect, supporting the MRS as a composite marker encompassing both known (insulin-related) and potentially novel aspects of obesogenic physiology.

Some limitations of this report should be recognized. First, the discovery and replication cohorts differ on risk factors such as ethnicity and lifestyle, thus the participants may have important biological differences (e.g. ΔBMI is not correlated with baseline BMI or WHR in FHS, but shows significant association in MCDS). While we were able to replicate the MRS despite these caveats, future replication in additional cohorts could yield a better estimate of the predictive power of MRS on weight gain across different settings. Next, by excluding samples with metabolic diseases (T2D, CVD, and renal disease) at baseline, we may have introduced selection bias, whereby subsetting data using a variable caused by two independent variables induces an association between them. Nevertheless, since these diseases are known to cause drastic changes in the metabolome, it was prudent to exclude them to reduce the risk of capturing a skewed profile. In addition, a similar bias may be caused by the fact that we needed to subtract out (i.e. “condition on”) baseline BMI in order to calculate ΔBMI, therefore potentially inducing associations between ΔBMI or MRS and other variables correlated with baseline BMI. This bias may contribute to our seeming paradoxical observation that the MRS is correlated with glycemic measures and negatively predictive of T2D. However, we note that (1) the MRS is more strongly associated with several glycemic variables compared to with baseline BMI (**S4 Table**) and (2) the MRS is independent from baseline BMI in conditional analysis to predict T2D in MCDS (**S8 Table**). These results provide some evidence that the association between the MRS and insulin response/T2D is not entirely a statistical artifact induced by our definition of ΔBMI. Finally, while we observed little correlation between ΔBMI or MRS and lifestyle factors that measure energy intake and expenditure in this report, lifestyle data were not available at every visit for both cohorts, and our investigations were also limited by the methods used to assess lifestyle in these cohorts. Therefore, future investigation is required to determine if the lack of correlation is a reflection of limited sample size and/or data availability. Additional data types (e.g. dietary quality measures or calorimetry-derived metabolic rate) may also help us assess if and how the MRS is linking imbalance in energy metabolism to weight gain.

In conclusion, we have systematically constructed an MRS that is a generalizable weight gain predictor and is independent from traditional risk factors. Interestingly, we found the MRS to be a strong marker for insulin sensitivity (even though they independently contribute to weight gain prediction) and showed that individuals with higher MRS have lower risk of developing T2D in one cohort, indicating that it may be more clinically important to track individuals with lower MRS who are less likely to gain weight but more metabolically unhealthy with respect to T2D. We were not able to test association of MRS with other obesity-associated outcomes, such as cancer, which may be related to unchecked weight gain and anabolic effects of insulin. Overall, our study showcases the power of leveraging metabolite profiling to elucidate and identify the role of intrinsic and extrinsic factors on weight change. Further studies of metabolite profiling and weight gain are required to replicate and expand these findings in independent cohorts. For instance, untargeted profiling methods may be used to discover many more unknown metabolites predictive of weight gain, which can greatly improve the power of the MRS model. Another critical future direction is to leverage genetic data to use Mendelian randomization approaches to establish if and how the MRS is capturing the causal biology of weight gain.

## Supporting information

S1 Fig Schematic overview for constructing, validating, and understanding the MRS for weight gain. The MRS was constructed using FHS data and tested for replication in MCDS. After validating that the MRS was associated with ΔBMI in both cohorts, we used phenotype and genetic data from both cohorts to study the relationships between MRS and various obesity-related risk factors, future T2D outcome, and genetic variants.
(PDF)

S2 Fig Sample relatedness in FHS. **(a)** Distribution of pedigree size (left) and number of individuals in pedigrees of different sizes (n = 1,508; right). **(b)** Distribution of identity by descent measure (PI_HAT) for all pairs of individuals with genetic data (n = 1,349; left) or subset of pairs with PI_HAT > 0.05 (right). PI_HAT was calculated using 92,210 independent genetic markers using PLINK (v1.9).
(PDF)

S3 Fig Comparison of ΔBMI-metabolite association statistics derived using simple linear model (LM) vs. linear mixed model (LMM) in FHS. Effect size estimates **(a)** and negative log_10_ *p*-value **(b)** of the 183 metabolites calculated using each approach are plotted. R^2^, squared correlation between the LM and LMM statistics.
(PDF)

S4 Fig Summary of cross validation results for the stepwise regression procedure in FHS. 100 repeated 10-fold cross validations were performed to determine the optimal direction of variable selection (DIRECTION: forward, backward, or both/bidirectional) and model evaluation metric (METRIC: AIC or BIC) for building a stepwise MRS model for predicting ΔBMI. R^2^ **(a)** and root-mean-squared error **(b)** statistics across the 100 cross validations are plotted as notched boxplots, with the notches indicating 95% confidence interval of the median.
(PDF)

S5 Fig Clustered correlation heat map of all 183 metabolites analyzed in FHS. The 8 MRS metabolites and the other 34 ΔBMI-associated (*p* < 0.05) metabolites are labeled in red and blue, respectively. CE, Cholesteryl esters; DAG, diacylglyceride; F1P/F6P/G1P/G6P, fructose-1- phosphate/fructose-6-phosphate/glucose-1-phosphate/glucose-6-phosphate; LPC, lysophosphatidylcholine; LPE, lysophosphatidylethanolamine; PC, phosphatidylcholine; SM, sphingomyelin; TAG, triacylglyceride.
(PDF)

S6 Fig Density histogram of pairwise correlation between the 42 ΔBMI-associated metabolites (“Nominal”) vs. the subset of 8 metabolites included in the MRS (“MRS”) in FHS. Dashed vertical lines indicate mean correlation of each group.
(PDF)

S7 Fig ΔBMI vs. MRS for FHS (red) and MCDS (blue) samples. Linear regression lines (with shaded 95% confidence regions) and corresponding statistics (bottom right text labels) are shown.
(PDF)

S1 Table FHS and MCDS association statistics for ΔBMI-associated (*p* < 0.05) metabolites in FHS. * As a sensitivity analysis in FHS, LMM (linear mixed model) regression was performed to account for sample relatedness (see Methods); ** Nominally significant *p*-values are in bold. β, effect size estimate; SE, standard error of β; CI 2.5—CI 97.5, 95% confidence interval for β; LMM R^2^ (i.e. proportion of variance explained) estimated as: β^2^*var(Metabolite)/var(ΔBMI); Directional Consistency, whether the directions of effect in FHS and MCDS agree with each other; TAG, triacylglyceride; SM, sphingomyelin; PC, phosphatidylcholine; DAG, diacylglyceride; LPC, lysophosphatidylcholine; CE, cholesteryl ester.
(XLSX)

S2 Table Correlation and correlation *p*-value between the 8 MRS metabolites in FHS. Correlation (red) is shown in upper left triangle and correlation *p*-value (blue) is shown in lower right triangle. Metabolites are tabulated in order to match Fig 1 heat map. Nominally significant *p*-values are in bold.
(XLSX)

S3 Table Association between ΔBMI and MRS with adjustment for obesity-related risk factors. * Association Model: Y ~ X indicates linear regression with Y as dependent variable and X as independent variable(s). ** Nominally significant *p*-values are in bold. N, number of samples; β, effect size estimate; SE, standard error of β; CI 2.5—CI 97.5, 95% confidence interval for β; R^2^, proportion of variance explained, partial R^2^ is shown for models with multiple independent variables (see Methods).
(XLSX)

S4 Table Association between ΔBMI or MRS and obesity-related risk factors. * All risk factors were assessed at or prior to baseline, except for "Quit Smoking" and “ΔPA” (see Methods). ** Nominally significant *p*-values are in bold. Greyed out rows indicate that data were not available. N, number of samples; β, effect size estimate; SE, standard error of β; CI 2.5—CI 97.5, 95% confidence interval for β.
(XLSX)

S5 Table Association between MRS or MRS metabolites and dietary fats in FHS. * Nominally significant *p*-values are in bold. N = 1387 for all analyses. β, effect size estimate; SE, standard error of β; CI 2.5—CI 97.5, 95% confidence interval for β; TAG, triacylglyceride; SM, sphingomyelin.
(XLSX)

S6 Table Association between MRS metabolites and baseline anthropometric or glycemic measures. * Nominally significant *p*-values are in bold. N, number of samples; β, effect size estimate; SE, standard error of β; CI 2.5—CI 97.5, 95% confidence interval for β; TAG, triacylglyceride; SM, sphingomyelin.
(XLSX)

S7 Table Association between future T2D status and baseline anthropometric or glycemic risk factors. * Nominally significant *p*-values are in bold. N total, number of samples; N cases, number of T2D cases (by follow-up); OR, odds ratio; CI 2.5—CI 97.5, 95% confidence interval for OR.
(XLSX)

S8 Table Association between future T2D status and MRS with adjustment for baseline anthropometric or glycemic measures. * Association Model: Y ~ X indicates logistic regression with Y as dependent variable and X as independent variable(s). ** Nominally significant *p*-values are in bold. N total, number of samples; N cases, number of T2D cases (by follow-up); OR, odds ratio; CI 2.5—CI 97.5, 95% confidence interval for OR.
(XLSX)

S9 Table List of GWAS catalog associations (*p* < 5E-8) that overlap (± 50kb) with suggestive MRS lead variants. PMID, PubMed ID of study that reported the association; Chr, chromosome; Position_hg38, hg38 genomic position; β or OR, effect size or odds ratio estimate for the effect allele.
(XLSX)

S10 Table Genetic loci reaching suggestive significance (*p* < 1E-6) in MRS metabolite GWAS meta-analysis. * Genome-wide significant *p*-values are in bold. ** Loci boundaries were defined by linkage disequilibrium proxies (< = 500kb and r^2^ > 0.1) with minimum and maximum chromosomal positions. Chr, chromosome; Position, hg19 genomic position; EA, effect allele (i.e. metabolite-increasing allele); OA, other allele; EAF, effect allele frequency; β, effect size estimate; SE, standard error of β; R^2^, proportion of variance explained, estimated as: β^2^*2f(1-f), where f = EAF; TAG, triacylglyceride; SM, sphingomyelin.
(XLSX)

S1 Text Details of FHS genetic data. (DOCX)

S2 Text Details of MCDS genetic data. (DOCX)

S3 Text Missing value imputation for metabolite data. (DOCX)
