# Supplementary material for: Using metabolite profiling to construct and validate a metabolite risk score for predicting future weight gain
Source: PLoS One. 2019 Sep 27;14(9):e0222445. doi: 10.1371/journal.pone.0222445 (PMC6764659; doi:10.1371/journal.pone.0222445)
Supplement: S1 Fig — The MRS was constructed using FHS data and tested for replication in MCDS. After validating that the MRS was associated with ΔBMI in both cohorts, we used phenotype and genetic data from both cohorts to study the relationships between MRS and various obesity-related risk factors, future T2D outcome, and genetic variants. (PDF) [file pone.0222445.s001.pdf]

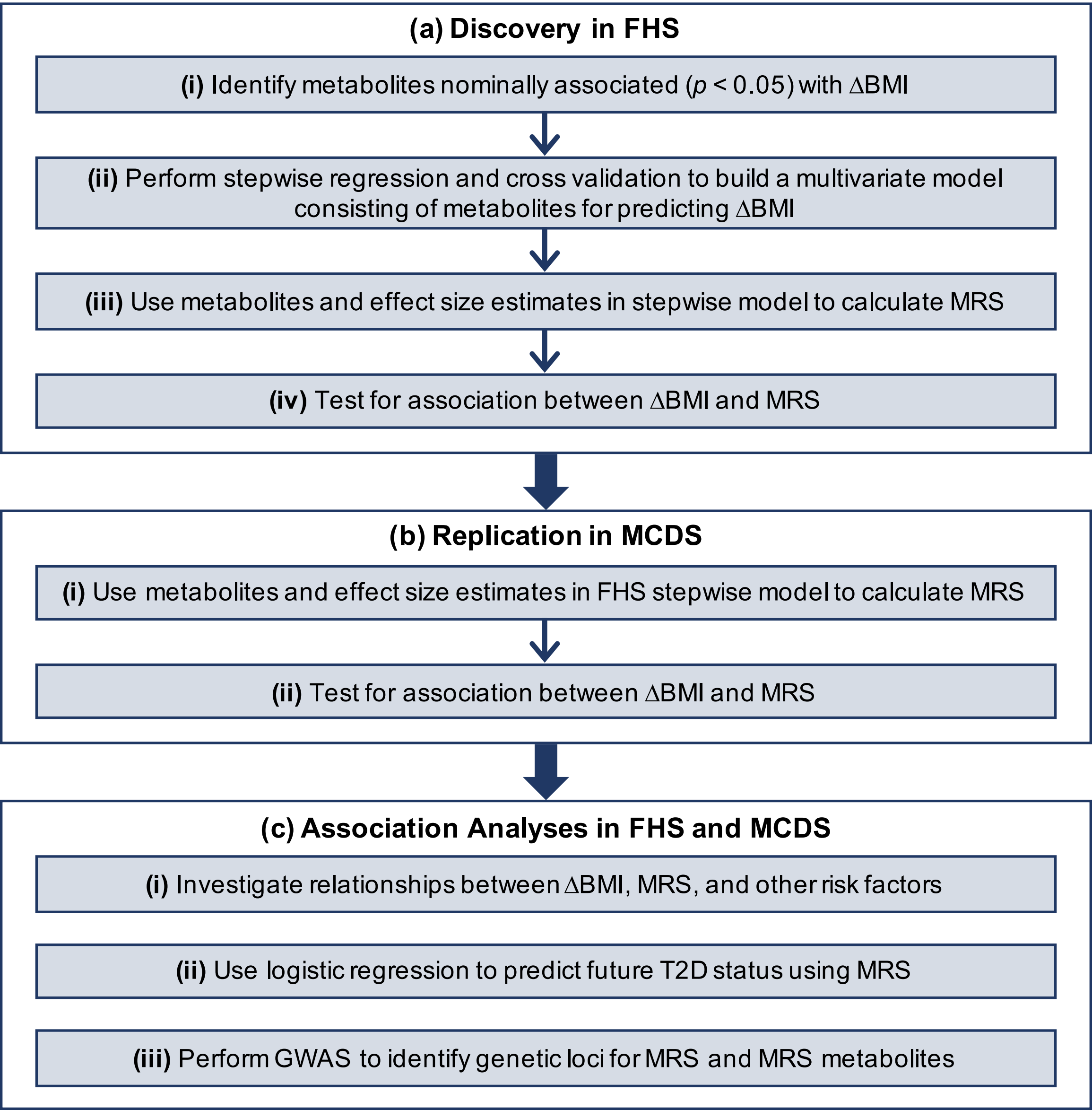
(a) Discovery in FHS
(i) Identify metabolites nominally associated ($p < 0.05$) with ΔBMI
(ii) Perform stepwise regression and cross validation to build a multivariate model consisting of metabolites for predicting ΔBMI
(iii) Use metabolites and effect size estimates in stepwise model to calculate MRS
(iv) Test for association between ΔBMI and MRS
(b) Replication in MCDS
(i) Use metabolites and effect size estimates in FHS stepwise model to calculate MRS
(ii) Test for association between ΔBMI and MRS
(c) Association Analyses in FHS and MCDS
(i) Investigate relationships between ΔBMI, MRS, and other risk factors
(ii) Use logistic regression to predict future T2D status using MRS
(iii) Perform GWAS to identify genetic loci for MRS and MRS metabolites
