# Supplementary figures and images for: Using metabolite profiling to construct and validate a metabolite risk score for predicting future weight gain
Source: PLoS One. 2019 Sep 27;14(9):e0222445. doi: 10.1371/journal.pone.0222445 (PMC6764659; doi:10.1371/journal.pone.0222445)

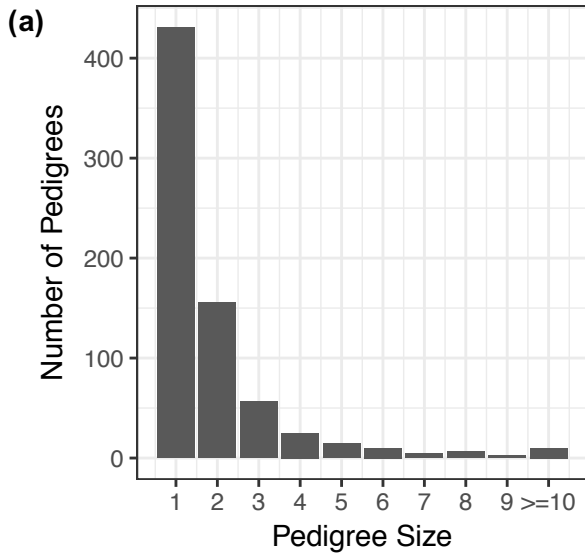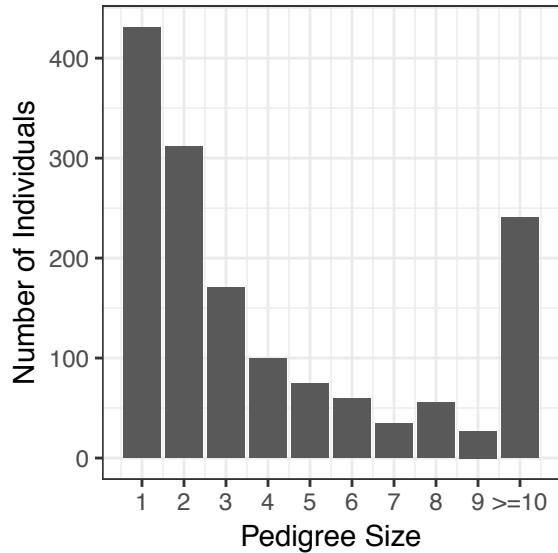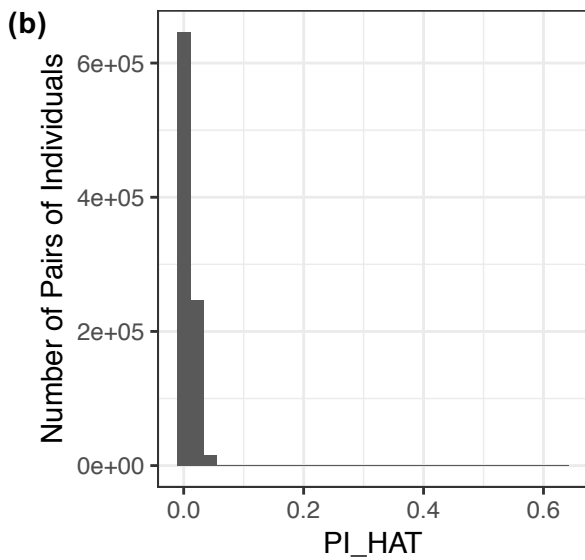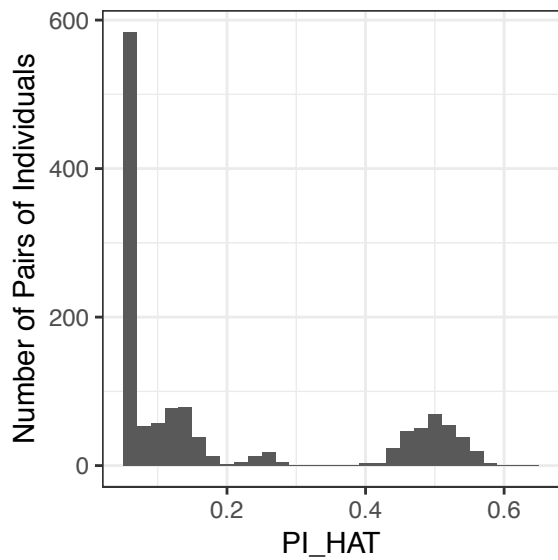

Supplement: S2 Fig — (a) Distribution of pedigree size (left) and number of individuals in pedigrees of different sizes (n = 1,508; right). (b) Distribution of identity by descent measure (PI_HAT) for all pairs of individuals with genetic data (n = 1,349; left) or subset of pairs with PI_HAT > 0.05 (right). PI_HAT was calculated using 92,210 independent genetic markers using PLINK (v1.9). (PDF) [file pone.0222445.s002.pdf]

(a)

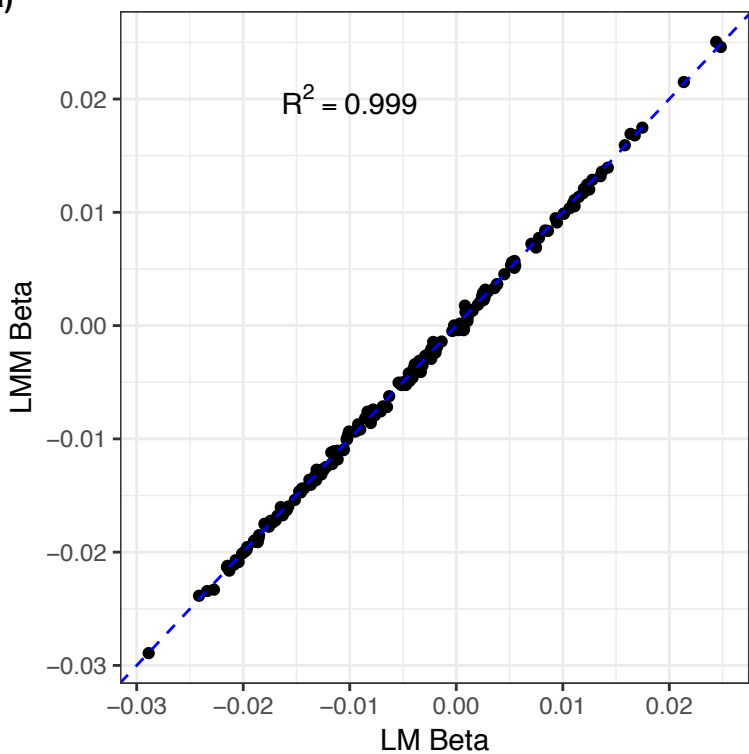

(b)

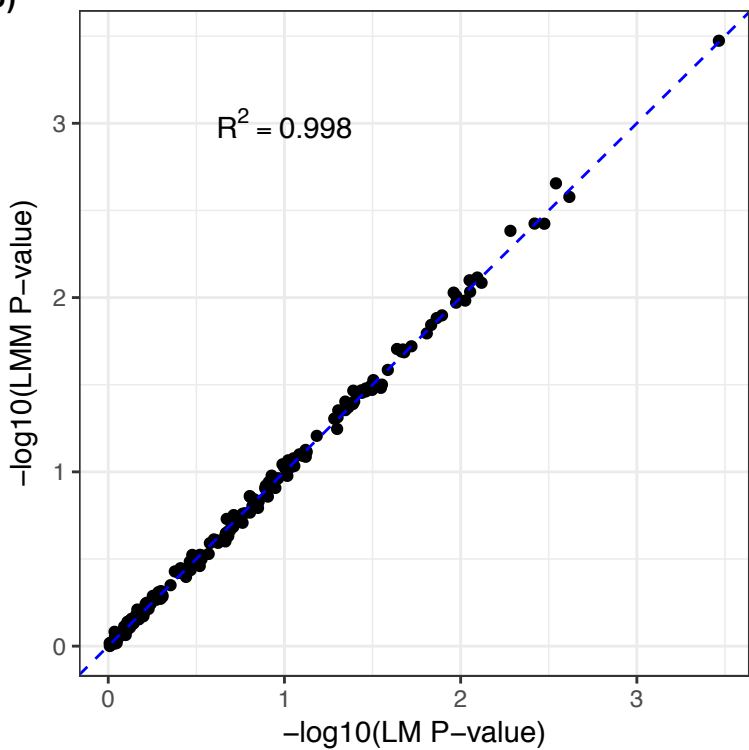

Supplement: S3 Fig — Effect size estimates (a) and negative log10 p-value (b) of the 183 metabolites calculated using each approach are plotted. R2, squared correlation between the LM and LMM statistics. (PDF) [file pone.0222445.s003.pdf]

(a)

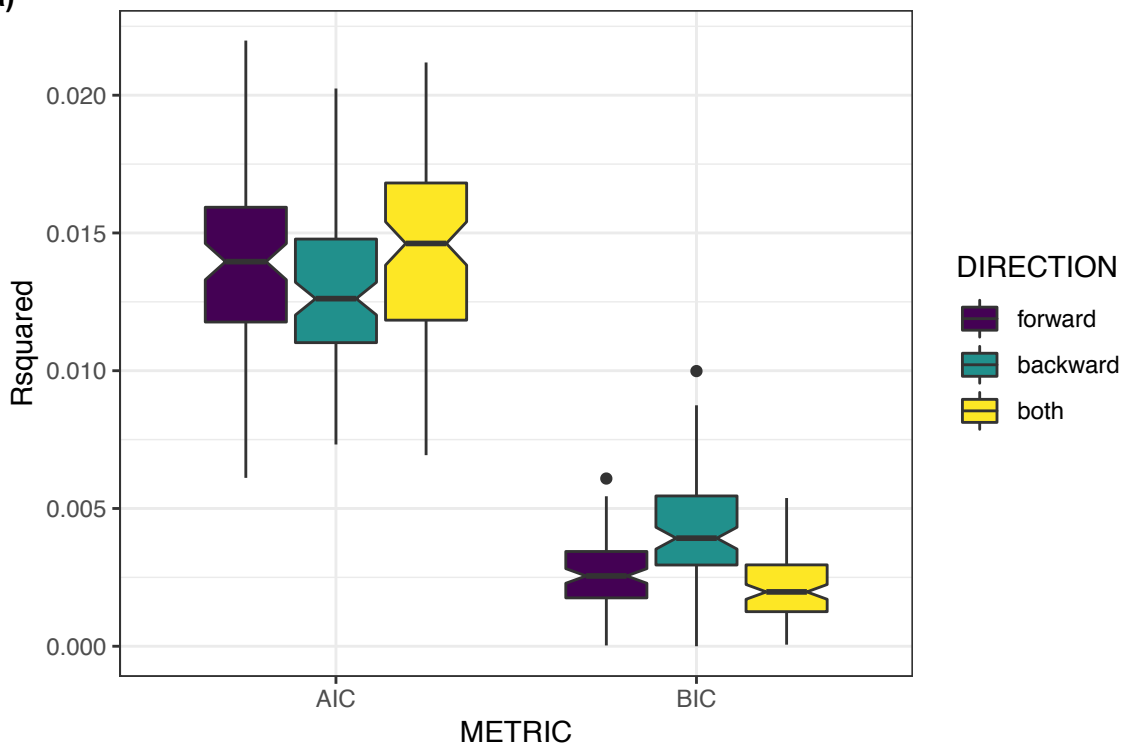

(b)

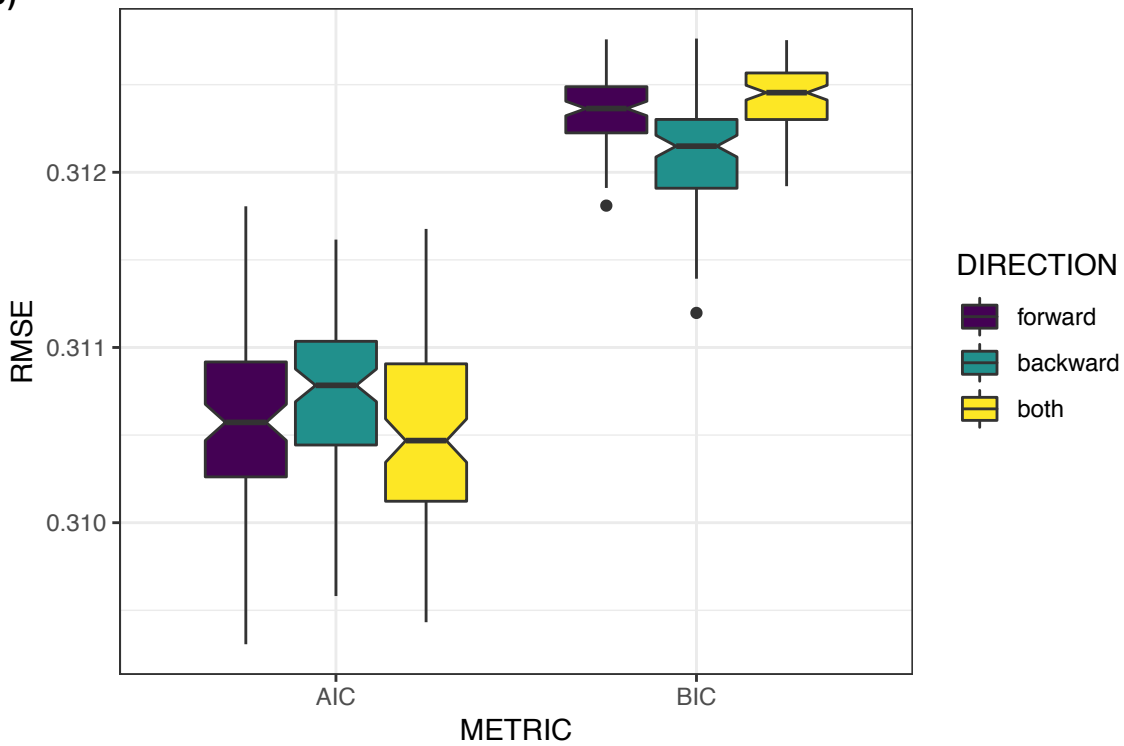

Supplement: S4 Fig — 100 repeated 10-fold cross validations were performed to determine the optimal direction of variable selection (DIRECTION: forward, backward, or both/bidirectional) and model evaluation metric (METRIC: AIC or BIC) for building a stepwise MRS model for predicting ΔBMI. R2 (a) and root-mean-squared error (b) statistics across the 100 cross validations are plotted as notched boxplots, with the notches indicating 95% confidence interval of the median. (PDF) [file pone.0222445.s004.pdf]

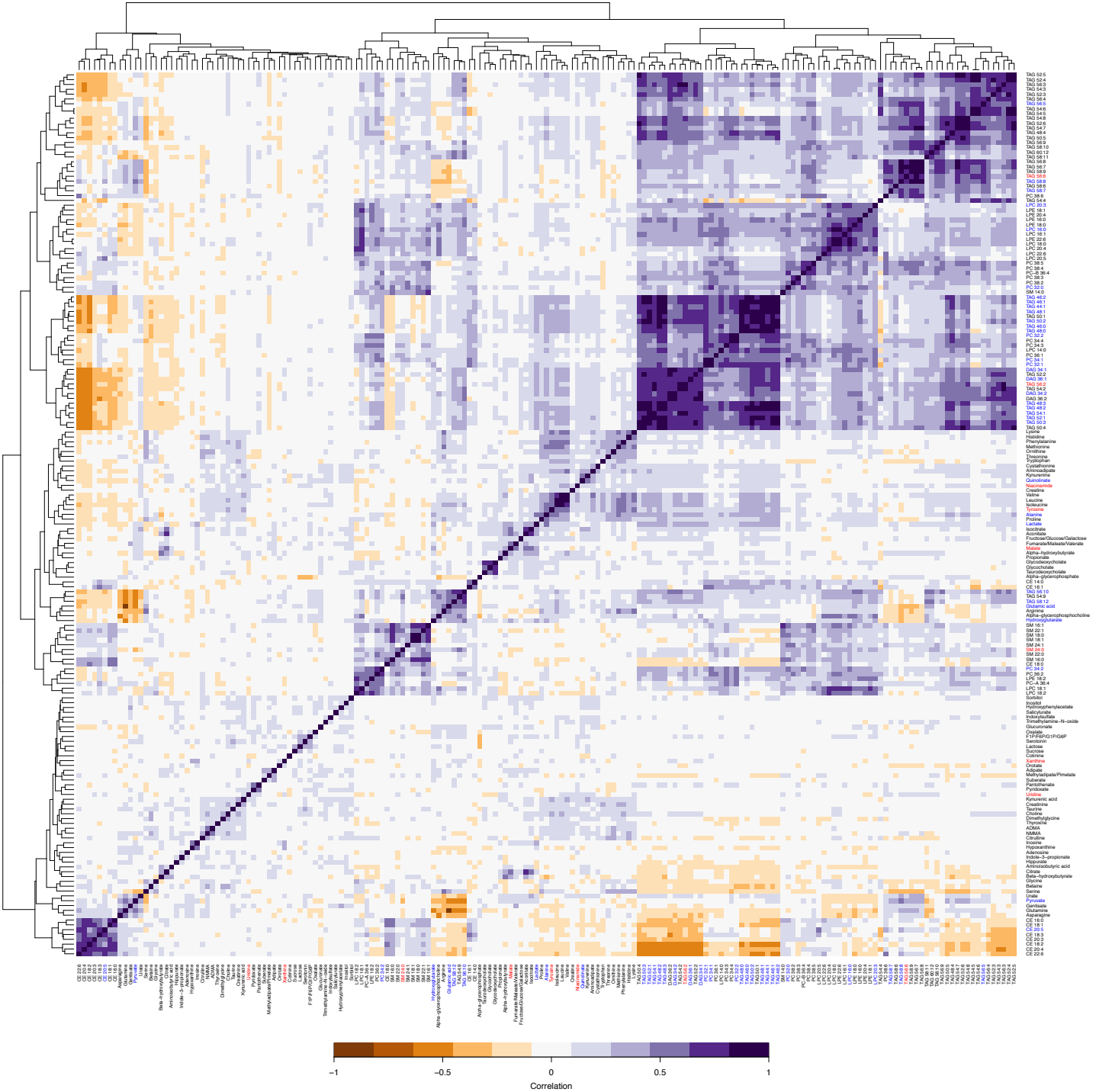

Supplement: S5 Fig — The 8 MRS metabolites and the other 34 ΔBMI-associated (p < 0.05) metabolites are labeled in red and blue, respectively. CE, Cholesteryl esters; DAG, diacylglyceride; F1P/F6P/G1P/G6P, fructose-1- phosphate/fructose-6-phosphate/glucose-1-phosphate/glucose-6-phosphate; LPC, lysophosphatidylcholine; LPE, lysophosphatidylethanolamine; PC, phosphatidylcholine; SM, sphingomyelin; TAG, triacylglyceride. (PDF) [file pone.0222445.s005.pdf]

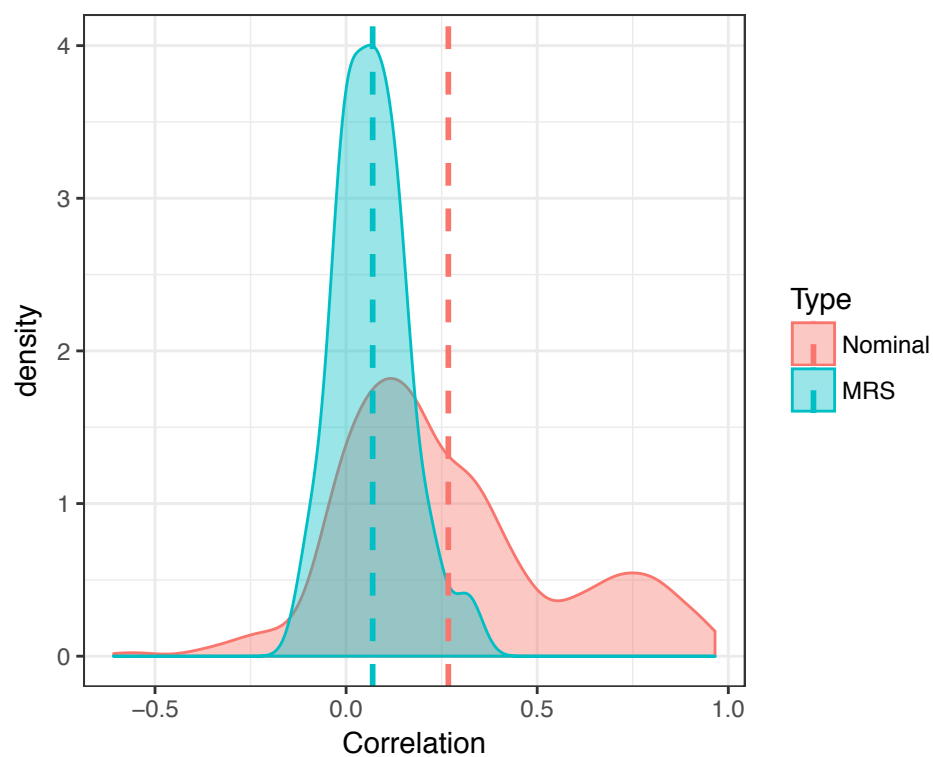

Supplement: S6 Fig — Dashed vertical lines indicate mean correlation of each group. (PDF) [file pone.0222445.s006.pdf]

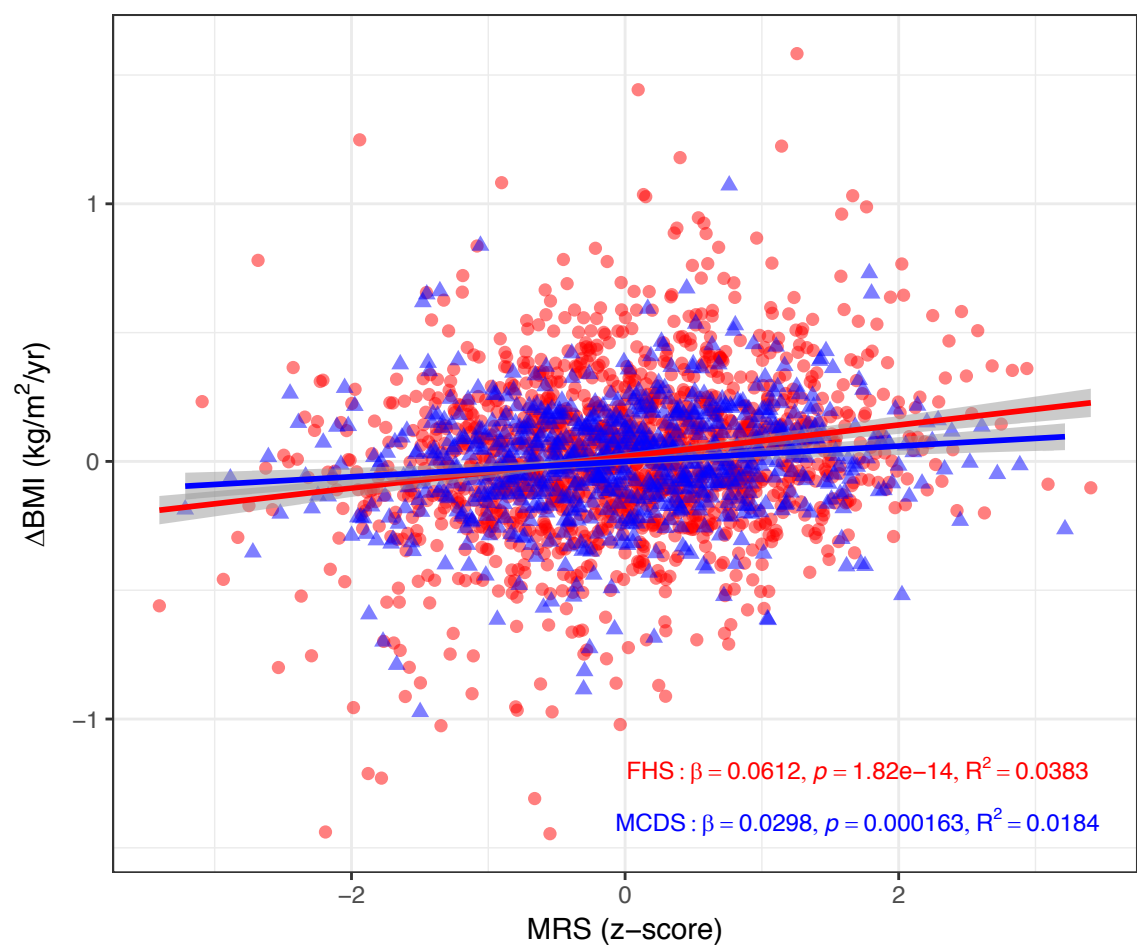

Supplement: S7 Fig — Linear regression lines (with shaded 95% confidence regions) and corresponding statistics (bottom right text labels) are shown. (PDF) [file pone.0222445.s007.pdf]
