# Supplementary material for: Using metabolite profiling to construct and validate a metabolite risk score for predicting future weight gain
Source: PLoS One. 2019 Sep 27;14(9):e0222445. doi: 10.1371/journal.pone.0222445 (PMC6764659; doi:10.1371/journal.pone.0222445)
Supplement: S1 Text — (DOCX) [file pone.0222445.s018.docx]

**S1 Text. Details of FHS genetic data.**

Quality control: A stringent quality control protocol was applied to the FHS genome-wide genotyping data retrieved from dbGaP. The protocol includes removal of subjects with discordant genetics and reported sex, samples that have > 2% missing SNPs, SNPs that have minor allele frequencies < 1%, SNPs that have > 5% missing rate, and samples that have extreme heterozygosity (± 4 SD). The SNP annotations for chromosome and base-pair positions were set to the coordinates of hg19 (GRCh37) via the liftOver tool ^1^. Pairwise IBD/IBS were calculated and individuals that have excessive matching with other individuals were removed. Principal components analysis was performed with SMARTPCA ^2^ and each study was projected onto HapMap v3 ^3^ space. Samples that did not cluster with the expected HapMap population were removed. SNPs that had excessive plate effects (p < 1 × 10^−7^) or excessive deviation from Hardy-Weinberg equilibrium (p < 1 × 10^−7^) were dropped. The QC protocol was performed with PLINK (v1.07) ^4^ and custom R and PERL scripts.

Phasing and imputation: Genotypes were phased with SHAPEIT2 (v2.644) ^5^ and imputed with IMPUTE2 (v2.3) ^6^. FHS was imputed using 1000 Genomes phase 1 v3 cosmopolitan reference panel ^7^. The imputation panel consists of approximately 22 million variants (SNPs, insertions, and deletions).

FHS dbGaP Acknowledgement: The Framingham Heart Study is conducted and supported by the National Heart, Lung, and Blood Institute (NHLBI) in collaboration with Boston University (Contract No. N01-HC-25195 and HHSN268201500001I). Funding for SHARe Affymetrix genotyping was provided by NHLBI Contract N02-HL-64278. SHARe Illumina genotyping was provided under an agreement between Illumina and Boston University. Funding for Affymetrix genotyping of the FHS Omni cohorts was provided by Intramural NHLBI funds from Andrew D. Johnson and Christopher J. O’Donnell. Additional funding for SABRe was provided by Division of Intramural Research, NHLBI, and Center for Population Studies, NHLBI. Funding support for the Framingham Food Frequency Questionnaire dataset was provided by ARS Contract #53-3k06-5-10, ARS Agreement #’s 58-1950-9-001, 58-1950-4-401 and 58-1950-7-707. Funding support for the Framingham Metabolomics (HILIC- Installment 1-3) dataset was provided by NIH grant R01 DK081572. This manuscript was not prepared in collaboration with investigators of the Framingham Heart Study and does not necessarily reflect the opinions or views of the Framingham Heart Study, Boston University, or NHLBI.

**References**

1. Fujita, P.A., B. Rhead, A.S. Zweig, A.S. Hinrichs, D. Karolchik, M.S. Cline, M. Goldman, G.P. Barber, H. Clawson, A. Coelho, M. Diekhans, T.R. Dreszer, B.M. Giardine, R.A. Harte, J. Hillman-Jackson, F. Hsu, V. Kirkup, R.M. Kuhn, K. Learned, C.H. Li, L.R. Meyer, A. Pohl, B.J. Raney, K.R. Rosenbloom, K.E. Smith, D. Haussler, and W.J. Kent, *The UCSC Genome Browser database: update 2011.* Nucleic Acids Res, 2011. 39(Database issue): p. D876-82. PMCIDPMC3242726

2. Price, A.L., N.J. Patterson, R.M. Plenge, M.E. Weinblatt, N.A. Shadick, and D. Reich, *Principal components analysis corrects for stratification in genome-wide association studies.* Nat Genet, 2006. 38(8): p. 904-9.

3. International HapMap, C., *A haplotype map of the human genome.* Nature, 2005. 437(7063): p. 1299-320. PMCIDPMC1880871

4. Purcell, S., B. Neale, K. Todd-Brown, L. Thomas, M.A. Ferreira, D. Bender, J. Maller, P. Sklar, P.I. de Bakker, M.J. Daly, and P.C. Sham, *PLINK: a tool set for whole-genome association and population-based linkage analyses.* Am J Hum Genet, 2007. 81(3): p. 559-75. PMCIDPMC1950838

5. Delaneau, O., J.F. Zagury, and J. Marchini, *Improved whole-chromosome phasing for disease and population genetic studies.* Nat Methods, 2013. 10(1): p. 5-6.

6. Howie, B., C. Fuchsberger, M. Stephens, J. Marchini, and G.R. Abecasis, *Fast and accurate genotype imputation in genome-wide association studies through pre-phasing.* Nat Genet, 2012. 44(8): p. 955-9. PMCID3696580

7. Genomes Project, C., G.R. Abecasis, A. Auton, L.D. Brooks, M.A. DePristo, R.M. Durbin, R.E. Handsaker, H.M. Kang, G.T. Marth, and G.A. McVean, *An integrated map of genetic variation from 1,092 human genomes.* Nature, 2012. 491(7422): p. 56-65. PMCIDPMC3498066
