# Supplementary material for: Using metabolite profiling to construct and validate a metabolite risk score for predicting future weight gain
Source: PLoS One. 2019 Sep 27;14(9):e0222445. doi: 10.1371/journal.pone.0222445 (PMC6764659; doi:10.1371/journal.pone.0222445)
Supplement: S2 Text — (DOCX) [file pone.0222445.s019.docx]

**S2 Text. Details of MCDS genetic data.**

MCDS is part of the SIGMA consortium, for which genotyping of the participants using the Illumina OMNI2.5 array ^1^, exome sequencing ^2^, and exome array ^3^ have been described previously. Genotype imputation of SIGMA samples have also been described previously ^3^. Briefly, we first combined data across the three genotyping platforms to build two datasets. One dataset was comprised of 4,478 samples that had been genotyped by exome chip and OMNI2.5 (MCDS: n = 59). The other dataset comprised of 3,732 samples genotyped by exome chip, OMNI2.5, and exome sequencing (MCDS: n = 529). For each dataset, we filtered for variants with MAF > 0.001, phased the data using SHAPEIT2 (v2.5) ^4^, and performed imputation using the 1000 Genomes reference panel (phase 3, release June 2014) and IMPUTE2 (v2.3.2) ^5^. We merged the two imputed datasets into one dataset and included genotyping backbone/platform as a covariate when performing GWAS.

**References**

1. SIGMA T2D Consortium, A.L. Williams, S.B. Jacobs, H. Moreno-Macias, A. Huerta-Chagoya, C. Churchhouse, C. Marquez-Luna, H. Garcia-Ortiz, M.J. Gomez-Vazquez, N.P. Burtt, C.A. Aguilar-Salinas, C. Gonzalez-Villalpando, J.C. Florez, L. Orozco, C.A. Haiman, T. Tusie-Luna, and D. Altshuler, *Sequence variants in SLC16A11 are a common risk factor for type 2 diabetes in Mexico.* Nature, 2014. 506(7486): p. 97-101. PMCIDPMC4127086

2. Sigma Type 2 Diabetes Consortium, K. Estrada, I. Aukrust, L. Bjorkhaug, N.P. Burtt, J.M. Mercader, H. Garcia-Ortiz, A. Huerta-Chagoya, H. Moreno-Macias, G. Walford, J. Flannick, A.L. Williams, M.J. Gomez-Vazquez, J.C. Fernandez-Lopez, A. Martinez-Hernandez, S. Jimenez-Morales, F. Centeno-Cruz, E. Mendoza-Caamal, C. Revilla-Monsalve, S. Islas-Andrade, E.J. Cordova, X. Soberon, M.E. Gonzalez-Villalpando, E. Henderson, L.R. Wilkens, L. Le Marchand, O. Arellano-Campos, M.L. Ordonez-Sanchez, M. Rodriguez-Torres, R. Rodriguez-Guillen, L. Riba, L.A. Najmi, S.B. Jacobs, T. Fennell, S. Gabriel, P. Fontanillas, C.L. Hanis, D.M. Lehman, C.P. Jenkinson, H.E. Abboud, G.I. Bell, M.L. Cortes, M. Boehnke, C. Gonzalez-Villalpando, L. Orozco, C.A. Haiman, T. Tusie-Luna, C.A. Aguilar-Salinas, D. Altshuler, P.R. Njolstad, J.C. Florez, and D.G. MacArthur, *Association of a low-frequency variant in HNF1A with type 2 diabetes in a Latino population.* JAMA, 2014. 311(22): p. 2305-14. PMCIDPMC4425850

3. Mercader, J.M., R.G. Liao, A.D. Bell, Z. Dymek, K. Estrada, T. Tukiainen, A. Huerta-Chagoya, H. Moreno-Macias, K.A. Jablonski, R.L. Hanson, G.A. Walford, I. Moran, L. Chen, V. Agarwala, M.L. Ordonez-Sanchez, R. Rodriguez-Guillen, M. Rodriguez-Torres, Y. Segura-Kato, H. Garcia-Ortiz, F. Centeno-Cruz, F. Barajas-Olmos, L. Caulkins, S. Puppala, P. Fontanillas, A.L. Williams, S. Bonas-Guarch, C. Hartl, S. Ripke, G. Diabetes Prevention Program Research, K. Tooley, J. Lane, C. Zerrweck, A. Martinez-Hernandez, E.J. Cordova, E. Mendoza-Caamal, C. Contreras-Cubas, M.E. Gonzalez-Villalpando, I. Cruz-Bautista, L. Munoz-Hernandez, D. Gomez-Velasco, U. Alvirde, B.E. Henderson, L.R. Wilkens, L. Le Marchand, O. Arellano-Campos, L. Riba, M. Harden, P. Broad Genomics, S. Gabriel, T.D.G. Consortium, H.E. Abboud, M.L. Cortes, C. Revilla-Monsalve, S. Islas-Andrade, X. Soberon, J.E. Curran, C.P. Jenkinson, R.A. DeFronzo, D.M. Lehman, C.L. Hanis, G.I. Bell, M. Boehnke, J. Blangero, R. Duggirala, R. Saxena, D. MacArthur, J. Ferrer, S.A. McCarroll, D. Torrents, W.C. Knowler, L.J. Baier, N. Burtt, C. Gonzalez-Villalpando, C.A. Haiman, C.A. Aguilar-Salinas, T. Tusie-Luna, J. Flannick, S.B.R. Jacobs, L. Orozco, D. Altshuler, J.C. Florez, and S.T.D.G. Consortium, *A Loss-of-Function Splice Acceptor Variant in IGF2 Is Protective for Type 2 Diabetes.* Diabetes, 2017. 66(11): p. 2903-2914. PMCIDPMC5652606

4. Delaneau, O., J.F. Zagury, and J. Marchini, *Improved whole-chromosome phasing for disease and population genetic studies.* Nat Methods, 2013. 10(1): p. 5-6.

5. Howie, B., C. Fuchsberger, M. Stephens, J. Marchini, and G.R. Abecasis, *Fast and accurate genotype imputation in genome-wide association studies through pre-phasing.* Nat Genet, 2012. 44(8): p. 955-9. PMCID3696580
