# Supplementary material for: Using metabolite profiling to construct and validate a metabolite risk score for predicting future weight gain
Source: PLoS One. 2019 Sep 27;14(9):e0222445. doi: 10.1371/journal.pone.0222445 (PMC6764659; doi:10.1371/journal.pone.0222445)
Supplement: S3 Text — (DOCX) [file pone.0222445.s020.docx]

**S3 Text. Missing value imputation for metabolite data.**

We imputed missing values in metabolite data using the MICE (multivariate imputation by chained equations) R package (v2.25) ^1^. In brief, MICE is an iterative algorithm that loops through each variable with missing data to predict its values using other correlated variables as predictors in a regression model. Typically, to account for uncertainty, the algorithm is run multiple times to impute a dataset and the resulting imputations are either all carried into downstream analysis or averaged into a single imputation. For each of our datasets, we used the default MICE model (predictive mean matching) and settings to perform 5 imputation runs of 20 iterations each, and took the median of the separate imputations to create the final imputed dataset.

In order to assess imputation quality, we simulated metabolite data in which a subset of metabolites had their lowest or random values set to missing, imputed these values using MICE or alternative approaches (i.e. no imputation, or imputing missing values to be the minimum or median of each metabolite), and then calculated the correlation between the imputed and true values to evaluate the performance of each imputation approach. We also tested real data by using the MICE imputed datasets and datasets generated by the other approaches to identify metabolites associated with BMI. In both simulations and real analyses, we observed that MICE is a more flexible imputation approach that can accommodate different missingness patterns, which can lead to improved power in downstream analyses.

**References**

1. van Buuren, S. and K. Groothuis-Oudshoorn, *mice: Multivariate Imputation by Chained Equations in R.* Journal of Statistical Software, 2011. 45(3): p. 1-67.
